# Effect of vitamin D supplementation on inflammatory markers and total antioxidant capacity in breast cancer women using a machine learning technique

**DOI:** 10.37349/etat.2023.00180

**Published:** 2023-10-30

**Authors:** Marzieh Tahmasebi, Masoud Veissi, Seyed Ahmad Hosseini, Amir Jamshidnezhad

**Affiliations:** Indian Institute of Technology Guwahati, India; Nicola Normanno, Istituto Nazionale Tumori-IRCCS-Fondazione G. Pascale, Italy; ^1^Nutrition and Metabolic Diseases Research Center, Ahvaz Jundishapur University of Medical Sciences, Ahvaz 61357-15794, Iran; ^2^Department of Nutritional Sciences, School of Allied Medical Sciences, Ahvaz Jundishapur University of Medical Sciences, Ahvaz 61357-15794, Iran; ^3^Department of Health Informatics, School of Allied Medical Sciences, Ahvaz Jundishapur University of Medical Sciences, Ahvaz 61357-15794, Iran

**Keywords:** Vitamin D, oxidative stress, inflammation, breast cancer, machine learning, artificial neural network

## Abstract

**Aim:**

This study aimed to establish a learning system using an artificial neural network (ANN) to predict the effects of vitamin D supplementation on the serum levels of vitamin D, inflammatory factors, and total antioxidant capacity (TAC) in women with breast cancer.

**Methods:**

The data set of the current project was created from women with breast cancer who were referred to the Shafa State Hospital of Patients with Cancers in Ahvaz city, Iran. Modeling was implemented using the data set at the serum levels of vitamin D, tumor necrosis factor-α (TNF-α), transforming growth factor β (TGF-β), and TAC, before and after vitamin D_3_ supplement therapy. A prediction ANN model was designed to detect the effects of vitamin D_3_ supplementation on the serum level changes of vitamin D, inflammatory factors and TAC.

**Results:**

The results showed that the ANN model could predict the effect of vitamin D_3_ supplementation on the serum level changes of vitamin D, TNF-α, TGF-β1, and TAC with an accuracy average of 85%, 40%, 89.5%, and 88.1%, respectively.

**Conclusions:**

According to the findings of the study, the ANN method could accurately predict the effect of vitamin D_3_ supplementation on the serum levels of vitamin D, TNF-α, TGF-β1, and TAC. The results showed that the proposed ANN method can help specialists to improve the treatment process more confidently in terms of time and accuracy of predicting the influence of vitamin D supplementation on the factors affecting the progression of breast cancer (https://www.irct.ir/ identifier: IRCT2015090623924N1).

## Introduction

Breast cancer is a common disease and depends on several factors such as female gender, aging, family history of breast cancer, premature menstruation, belatedly menopause, advanced age at first live birth, genetic mutation, poor diet, obesity, smoking, alcohol consumption, and sedentary lifestyle, removal of the uterus, the experience of fibrocystic diseases and cancer of the uterus or ovaries and radiation exposure to the chest area [1, 2]. Recently, breast cancer has been considered an essential health issue worldwide and according to the reports of the World Health Organization has an annual upward trend of 1.8% to 2% [2, 3]. The prevalence of breast cancer among all women cancer is 23% [4]. In 2020, there were approximately 2.3 million women worldwide with breast cancer and 684,996 people died from the disease [5]. More than 1.6 percent of women’s deaths worldwide are due to this disease. Breast cancer is the most frequent cancer among women with a rate of 76% of prevalent malignancies among Iranian women, and is also increasing [1].

Oxidative stress and inflammation can cause and advance cancers [6, 7]. In cancer, the effect of oxidative damage has been proven. According to a study on cancer patients, the development and progression of tumors, angiogenesis, and metastasis can be the outcome of inflammation [8]. Cancer is not affected by the regulatory mechanisms of the body and can change the homeostasis of the body in favor of its progress [9]. High concentrations of serum inflammatory factors [tumor necrosis factor-α (TNF-α), transforming growth factor β (TGF-β)], and reduced total antioxidant capacity (TAC) are important risk factors for the development and progression of breast tumors [10, 11].

Many breast cancer patients are deficient in serum vitamin D, and vitamin D deficiency is associated with the progression of malignancies and increased mortality [10, 12, 13]. Since cancer is associated with inflammation, vitamin D can be helpful *versus* cancer by various mechanisms, such as anti-inflammatory effects and reducing the production of inflammatory cytokines [14, 15]. Studies have shown that vitamin D intake in people reduces inflammatory factors and thus the risk of cancer [13, 16]. Vitamin D has anti-inflammatory and anti-cancer effects [13, 17]. The antioxidant activity of vitamin D is also through the nuclear vitamin D receptor (VDR) by increasing the expression of antioxidant systems [18]. The VDR is a probable healing goal for breast cancer as a result of its abundance in all cells, and vitamin D is a ligand for its stimulation [19]. Numerous studies have reported the effects of vitamin D in cancer patients due to its antioxidant and anti-inflammatory activity [10, 20, 21]. In a double-blind controlled trial, Mohseni et al. [10] examined the effect of vitamin D supplementation on inflammatory markers (TNF-α, TGF-β) and TAC in women with breast cancer. The results of these studies revealed the positive effects of vitamin D supplementation on the aforesaid factors.

Breast cancer is a costly disease for patients, and the faster diagnosis of factors affecting the improvement of the treatment process helps to reduce mortality and recuperative patients’ quality of life [1, 22]. The use of accurate and cost-effective methods to improve the accuracy and speed of the treatment process is a fundamental subject owing to widespread mortality and the high expenses of treating this disease [23].

Recently, with the advancement of machine learning systems, the use of these techniques in medical predictions and diagnoses related to breast cancer has been enhanced [24]. The artificial intelligence methods can be used to understand the relationships between existing data and discover the laws governing them and can also be used to save many patients from ineffective and expensive diagnostic and therapeutic ways [24, 25].

An artificial neural network (ANN) is a common method of machine learning systems. ANNs are simulated based on the human brain, which uses the connection of artificial neurons through artificial synapses to understand input information [26]. The ANN consists of three layers. The first layer is the input layer for storing primary data. The second layer is the hidden layer for learning, and the third layer is the output layer for presenting results. The advantage of the ANN method is the ability to use fewer data and input by providing the best results to help overcome medical limitations [27–29]. The ANNs operate based on experience and have a special strength to analyze and understand relationships between data, then modeling and inference. The design of the ANN in analyzing the effects of clinical diets can be very advantageous [29].

ANN models showed suitable performance in predicting the effect of supplement therapy whereas in those studies, the experimental information and treatment intervention results were obtained from the clinical trial studies [23, 30, 31]. In these studies, the ANNs predicted, successfully the effect of supplementations on the inflammatory markers as the main factors in the models [23, 30].

As far as we know, no study has focused on predicting the effect of vitamin D_3_ supplementation in the recovery of breast cancer patients based on machine learning algorithms. Therefore, the main purpose of this study was to predict the effect of vitamin D_3_ supplementation on the serum level changes of vitamin D, inflammatory factors (TNF-α, TGF-β1), and TAC using an ANN.

## Materials and methods

### Dataset

This analytical study was conducted to predict the effect of vitamin D_3_ supplements received 50,000 IU/week (provided by Zahravi Pharm. Co., Tabriz, Iran), for a period of 8 weeks, on the serum levels of vitamin D, TNF-α, TGF-β1, and TAC. In this study, research factors were obtained from women with breast cancer who were referred to the Shafa Specialty Cancer Diseases Hospital in Ahvaz, Khouzestan, the center of the southwest state of Iran [10]. Blood samples were collected for measurement of serum 25-hydroxyvitamin D [25(OH)D] concentrations before and after the intervention. Enzyme-linked immunosorbent assay (ELISA) method was used for measuring the serum levels of TNF-α, TGF-β1, and TAC [ELISA kits of bioactive diagnostic GmbH Company (Homburg, Germany)].

The baseline characteristics of the clinical trial participants in this study are shown in Table 1. Inclusion criteria included patients with breast cancer in stage I–III of the disease, age 30–60 years, and not receiving vitamin D supplements in the last 3 months. Patients with metastatic breast cancer or a precedent of other cancers, those who had a history of chemotherapy, radiotherapy, and hormone therapy for any reason other than the present disease, and people with chronic diarrhea and malabsorption were excluded from the study. Other exclusion factors included the use of corticosteroids, the patient’s known inflammatory disorders (such as acute bacterial or viral infections), and autoimmune diseases. The sample size was calculated based on a study with a confidence level of 95% and a power of 80% [32].

**Table 1:** Baseline characteristics of the clinical trial participants

**Characteristics**	**Placebo**	**Treatment group, vitamin D**	***P***
Demographics (age, years old), mean (SD)	46.3 (9.5)	47.7 (8.0)	0.6
Ethnicity, %			
Arab	54	43.5	0.46
Fars	46	56.5	
Breast cancer stage, %			
I	33	27	0.50
II	42	43	
III	25	30	
Anthropometric, mean ± SD			
BMI	29.2 ± 6.3	30.2 ± 5.4	0.59
Waist circumference	103.5 ± 12.5	109.0 ± 11.2	0.12
Mean dietary intakes, mean ± SD			
Total energy intake, kcal/day	1,596 ± 528	1,848 ± 821	0.59
Total fat, g/day	67 ± 32	70 ± 32	0.59
Dietary calcium, mg/day	618 ± 308	843 ± 526	0.41
Dietary fiber, g/day	15 ± 7	18 ± 9	0.97
Dietary carotenoid intake, (μg/day)	4,743.74 ± 4,771.63	4,509.17 ± 3,890.52	0.77
Dietary vitamin C intake, (mg/day)	104.16 ± 79.14	108.80 ± 84.43	0.16
Dietary vitamin E intake, (mg/day)	30.70 ± 18.28	29.80 ± 15.36	0.53
Dietary selenium intake, (μg/day)	48.50 ± 29.77	46.90 ± 27.63	0.68

BMI: body mass index; SD: standard deviation

The mean serum levels of vitamin D, TNF-α, TGF-β1 and TAC before and after vitamin D_3_ supplementation are indicated in Table 2.

**Table 2:** Change in biomarkers of serum after supplementation of vitamin D in breast cancer patients

**Biomarkers**	**Placebo**			**Vitamin D**			**Absolute treatment effect^a^**		
	**Baseline**	**2 Months**	***P* ^c^**	**Baseline**	**2 Months**	***P* ^c^**	**Placebo**	**Vitamin D**	***P* ^b^**
25(OH)D (ng/mL)	15.3 ± 2.2	13.4 ± 2.2	0.59	28 ± 2.6	39 ± 3.5	0.004^*^	–1.9 ± 0.9	11 ± 3.1	0.001^*^
TNF-α (pg/mL)	32.6 ± 8	25.6 ± 3.2	0.19	13.4 ± 1.1	14.5 ± 1.6	0.96	7 ± 2.1	1.1 ± 2.1	0.18
TGF-β (pg/mL)	123.4 ± 9	133.8 ± 10	0.24	293.8 ± 48.8	288 ± 42.9	0.84	10.4 ± 7.1	–5.6 ± 33.4	0.64
TAC (U/mL)	45.2 ± 11.5	29.2 ± 8.3	0.001^*^	48.9 ± 13.3	63.5 ± 13.3	0.004^*^	–16 ± 8.4	14.6 ± 8.9	0.017^*^

^a^ Absolute treatment effect is the absolute change from baseline to follow-up in the treatment group minus the absolute change from baseline to follow-up in the placebo group; ^b^ *P* values for differences between the treatment and placebo groups; ^c^ *P* values for difference between baseline visit and post-intervention values; ^*^ *P* value < 0.05; values are mean ± SD

The prediction model was developed using the breast cancer serum biomarkers including serum levels of vitamin D, TNF-α, TGF-β1, and TAC before and after vitamin D_3_ supplementation. Moreover, patients’ demographic, anthropometric, and dietary information as the main features for supplement therapy in patients with breast cancer were selected [10]. Generally, the performance of the models was examined with random trials, including 188 rotation data in total training, validating, and testing execution phases for four serum levels predictor models. In this study, 90% of the data was used randomly to train the network, and the remnant was applied to determine the validation of the model in terms of its comprehensiveness and its ability to be generalized to other data.

### ANN

An ANN framework is composed of several layers, i.e., the input layers, the hidden layers, and the output layers. ANNs have various kinds, such as multilayer perceptron (MLP), radial basis function, Hopfield, etc. In this study, the MLP neural network, which is a common type of neural network, was used to discover the relationship between input and output variables. To implement the proposed model, Matrix Laboratory (MATLAB) software version 2015b was used. The proposed model simulated the process of supplement therapy to predict the behavior of vitamin D in terms of its impact on the mentioned factors among women with breast cancer.

**Figure 1:**
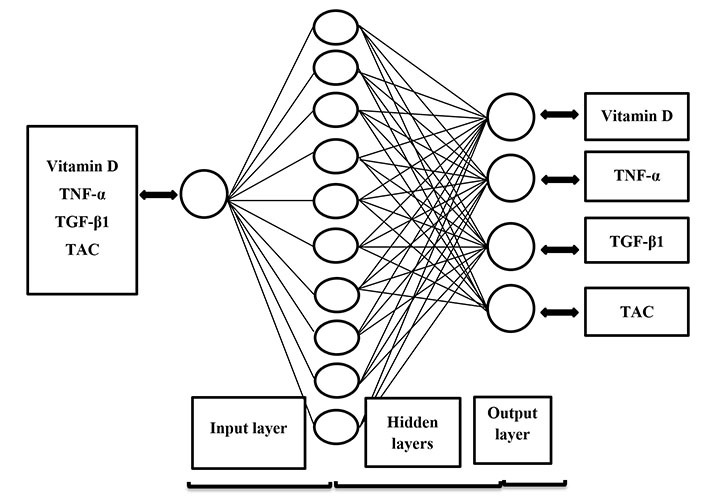
Structure of the ANN for predicting the effect of vitamin D on serum levels of vitamin D, TNF-α, TGF-β1, and TAC

Modeling was performed with the Neural Net Fitting Section of MATLAB environment. The data were entered consisting of four input data related to before the intervention and one output data related to after the intervention in four-stage separately. The structure of the neural network used is presented in Figure 1.

### Data analysis

The different architectures of the ANN were evaluated with diverse segmentation of data, disparate number of neurons and various training algorithms to achieve an optimal structure of the predictor model.

The data was divided into three forms: training, validation, and testing to adjust the network, measure the progress of the general network and evaluate the independent performance of the network, respectively. The disparate numbers of neurons were optimized by trial and error, including 5, 8, 10, 12, and 15 in various layers size. Moreover, the various training algorithms such as Levenberg-Marquardt, Bayesian Regularization, and Scaled Conjugate Gradient were also investigated to optimize the model performance.

Due to the limited number of available samples, the method of rotational placement was used with repetition in experiments of the proposed model to validate the model. Every designed model was evaluated 10 times using the training dataset. Moreover, the accuracy averages of all 10 runs in each pattern were calculated separately to specify the highest accuracy.

The performance of the ANNs models was evaluated using the mean squared error (MSE) through Equation 1. The MSE function was used to determine the error values in which the computing of three factors, including system output error, the square of the system error, and the mean of all the squared errors are possible.

The function formula is given below (Equation 1):

**Figure:**
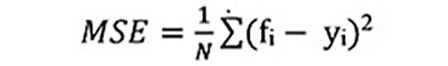


Where f_i_ is equivalent to the observed value, y_i_ is the corresponding predicted value for f_i_, and *N* is the number of observations [23].

## Results

In total, all experiments of the proposed models used 188 data (including 47 subjects in four experiments) to train, validate, and test phases using the random repetitive spin process to evaluate the models’ performance for serum levels of output factors in four predictors. Moreover, every predictor was run 10 times using the training dataset to evaluate the reliability of the model. After implementing and examining different models in a computer simulation, the optimal model with 10 neurons in the hidden layers with the Bayesian Regularization training algorithm was determined to predict the effect of the vitamin D supplementation.

The average accuracy rates obtained from 10 repetitions in the proposed model to predict the effects of vitamin D on four variables are presented in Table 3.

**Table 3:** Mean results for the effects of vitamin D on serum levels of vitamin D, TNF-α, TGF-β1, and TAC

**Variables**	**Mean (%)**		
	**Train**	**Test**	**Total**
Vitamin D	84.7 (1.55)	100 (0)	85.4 (0.66)
TNF-α	42.7 (17.24)	50 (80.62)	40 (16.11)
TGF-β1	90.3 (4.02)	100 (0)	89.5 (3.47)
TAC	90.6 (10.13)	100 (0)	88.1 (8.36)

Values are mean (SD)

**Figure 2:**
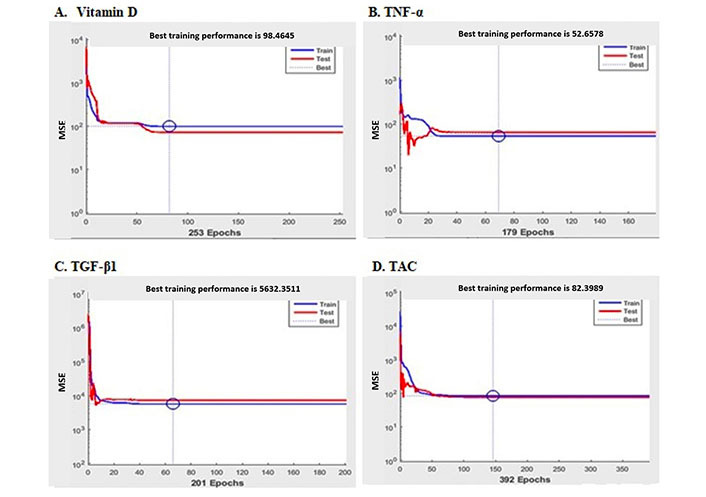
Neural network training performance

The performance of neural network in the training procedure to reduce gradually the estimated error level is shown in Figure 2. The level of accuracy and error of the implementation process is presented in these diagrams. The neural network training performance to predict the effect of vitamin D supplementation for variables of vitamin D, TNF-α, TGF-β1, and TAC is shown in Figure 2A, 2B, 2C, and 2D, respectively.

## Discussion

This study was conducted to predict the effect of vitamin D_3_ supplementation on the serum level changes of vitamin D, TNF-α, TGF-β1, and TAC in patients with breast cancer by ANN method in MATLAB environment. The results of this research have confirmed the precision of the ANN method to predict the effect of vitamin D supplementation on the variables. Nowadays, to improve the accuracy and speed of the patient’s treatment process, attention through the use of ANN techniques has increased. This project has been carried out in order to predict the effect of vitamin D supplementation on factors affecting breast cancer progression using the MLP neural network method, the most common and simplest type of neural network.

In our study, finding the best model for predicting the effect of vitamin D supplementation on the studied factors was a challenge. For this reason, the rotational placement and repetition methods were used in experiments related to modeling training, and different modes were brought together. The results of the study showed that the model can appropriately predict the effect of vitamin D supplementation on variables except for TNF-α.

### Vitamin D, inflammation and stress oxidative

Oxidative stress means an imbalance in the systems of oxidants and antioxidants, and inflammation can be the result of oxidative stress in the body. Oxidative stress and inflammation suppress the immune system and cause the formation and growth of tumors and thus the progression of cancer [33, 34]. Vitamin D is a prohormone that is actuated through hydroxylation pathways to create 1,25-dihydroxyvitamin D_3_ [35, 36]. Vitamin D, in addition to its well-known role in bone metabolism and calcium and phosphorus homeostasis, also has immune modulatory properties that play a role in cancer prevention and treatment [37]. Vitamin D has anti-inflammatory effects such as reducing the production of inflammatory cytokines [38]. VDRs have the aptitude to reduce the activity of nuclear factor-kappa B (NF-κB), a pro-inflammatory transcription factor [39, 40]. Vitamin D also exerts its antioxidant activity by binding to the VDR and adjusting gene expression. This vitamin increases the expression of antioxidant systems and restrains the release of reactive oxygen species [18].

### Vitamin D in cancer

An inverse relationship between the serum level of vitamin D and the development and progression of breast cancer has been shown; indeed, the reduction of the serum vitamin D level leads to an increase in the risk of relapse and premature death of these patients [41]. Several studies have shown the effect of vitamin D on intended factors in cancer patients [10, 20, 21]. Mohseni et al. [10] investigated the effects of vitamin D supplementation on serum vitamin D levels, inflammatory factors (TNF-α, TGF-β1), and TAC. Although the results of this study showed that vitamin D supplementation significantly increased the serum levels of vitamin D and TAC in patients with breast cancer, some studies have shown an insignificant relationship between vitamin D supplements and the risk of breast cancer according to the results of mammography density [42]. These conflicting results arrived from different factors in the studies such as the dose of vitamin D, the age of the studied population, and the condition of intervention. Moreover, in this study, the effects of vitamin D on the inflammatory factors were considered. In contrast, in previous studies, mammography density was reported as a positive factor for the risk of breast cancer. The changes in the serum levels of inflammatory factors, including TNF-α and TGF-β1, were not significant notwithstanding the reduction [10]. In other studies, Wesselink et al. [20] examined the association between circulating levels of vitamin D and inflammatory markers, including TNF-α, in patients with colorectal cancer. The results of this study showed a reverse correlation between the levels of vitamin D and TNF-α [20]. Another study by Qin et al. [21] examined the effect of vitamin D supplementation on serum TGF-β1 levels in women at high risk for breast cancer. The results showed that treatment with a dose of 400 IU of vitamin D for one month was effective in reducing the serum level of TGF-β1 [21].

In accordance with the above studies on the anti-inflammatory and antioxidant effects of vitamin D on the serum levels of vitamin D, inflammatory factors, and TAC in the treatment of cancer and due to the effect of increasing TNF-α and TGF-β1 [43, 44] and decreasing serum levels of TAC [45] and vitamin D in the progression of breast cancer, using the methods that can predict these effects accelerate the healing process and reduce mortality [34, 46]. In the current study, according to the existing correlation and its importance, some changes in the serum levels of the variables under the influence of vitamin D supplementation in patients with breast cancer were shown to be predictable.

### ANN in diseases

The application of artificial intelligence methods and clinical decision-making algorithms can accelerate the process of analyzing information related to patients, interpreting data, and predicting treatment results [47, 48], which also leads to the reduction of expenses related to the disease [49]. The results of the present study showed that the ANN can predict the effect of vitamin D_3_ supplementation on serum levels of vitamin D, TGF-β1, and TAC with high accuracy including 85.4%, 89.5%, and 88.1%, respectively. The average accuracy of the developed model for the TNF-α variable was 40%, which showed that there was not a significant correlation between the input variables and the TNF-α changes in the patient with vitamin D supplementation therapy.

Recently, studies have also been conducted to predict the effects of supplements using the ANN methods [23, 30, 31]. In a study conducted by Jamshidnezhad et al. [23] the effect of coenzyme Q10 (Q10) supplementation on inflammatory markers in women with breast cancer was investigated using a machine-learning predictive model. In this study, a MLP neural network was used, and the results showed that the use of an ANN could predict the levels of the impact of Q10 supplementation on inflammatory factors, including interleukin 6 (IL-6), IL-8, and vascular endothelial growth factor (VEGF) with an accuracy of 96%, 88%, and 92%, respectively [23].

Hosseini et al. [30] also conducted a study to predict the effect of saffron supplementation on the treatment of patients with asthma and allergies using the ANN method. In this study, the use of the ANN genetic algorithm method could accurately predict the levels of effects of saffron supplementation on anti-inflammatory factors [anti-heat shock protein (Hsp) and C-reactive protein (CRP)] in asthma with an accuracy of 96.5% and 98.9%. The results confirmed the effectiveness of the ANN in predicting the effect of the supplement on the treatment of patients with asthma [30].

In another study, Allahyari et al. [31] examined the prediction of response to vitamin D supplementation and changes in serum vitamin D levels based on neurophysiological factors in adolescent girls using the ANN. The results showed that the ANN algorithm with sigmoid transfer function could effectively predict the response to vitamin D supplementation with sensitivity and specificity between 60–70% [31].

In agreement with our project, studies have confirmed that machine learning methods such as ANN can predict the effect of different supplements on diseases. The application of these methods can help patients by saving time and economizing the costs during the periods of treatment. In general, studies have shown that we can use artificial intelligence algorithms instead of implementing old computational methods, to improve the performance of models in data analysis and understand patterns in them [50]. In the present study, we used the ANN and MATLAB software to predict the effect of vitamin D supplementation in women with breast cancer. The results of the present study highlight the importance of predicting the effect of vitamin D supplementation on the serum levels of vitamin D, inflammatory factors, and TAC in women with breast cancer using an ANN.

In the present study, the ANN was used to predict the effect of vitamin D supplementation on serum levels of vitamin D, inflammatory markers, and TAC in women with breast cancer. Given that the considered factors are effective in the progression of breast cancer, the results of this research can help to meliorate the treatment process of patients.

These techniques can help specialists improve the treatment procedure by predicting the effect of vitamin D supplementation on the factors affecting the progression of breast cancer in different people to prescribe vitamin D supplementation with greater confidence.

As study limitations, an exploration of more alternative machine learning approaches warrants consideration. Incorporating more data from diverse centers could augment result quality and real-world relevance. Furthermore, independent evaluation of treatment methodologies and their impacts on patients with cancer, alongside a deeper probe into drug effects through machine learning, beckon as promising avenues for future research in this domain.

## Abbreviations

ANN: artificial neural network
MATLAB: Matrix Laboratory
MLP: multilayer perceptron
MSE: mean squared error
TAC: total antioxidant capacity
TGF-β: transforming growth factor β
TNF-α: tumor necrosis factor-α
VDR: vitamin D receptor

## Declarations

### Copyright

© The Author(s) 2023.
